# A Preliminary Neural Network-Based Composite Method for Accurate Prediction of Enthalpies of Formation

**DOI:** 10.1021/acs.jctc.4c01351

**Published:** 2024-12-12

**Authors:** Gabriel César Pereira, Rogério Custodio

**Affiliations:** Instituto de Química, Universidade Estadual de Campinas Barão Geraldo, P.O. Box 6154, 13083-970 Campinas, São Paulo, Brazil

## Abstract

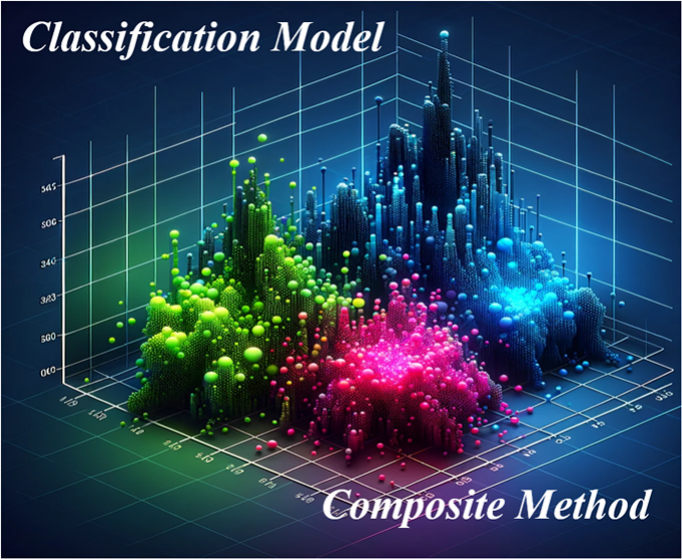

A composite method, named ANN-G3S, is introduced, adapting from G3S theory and employing distinct sets of multiplicative scale factors. An artificial neural network (ANN)-based classification model is utilized to select optimal sets of four scale factors for electronic correlation and basis set expansion terms in electronic systems. The correlation and basis set terms are scaled by four parameters, two for atoms and the other two for molecules. The ANN model is trained on the G3/05 test set to identify the best parameter set for each electronic system. To validate the method, 10% of the structures from the test set are randomly excluded from training and optimization, forming a separate validation set. The method demonstrates a mean deviation of 1.11 kcal mol^–1^ for the G3/05 set and 0.89 kcal mol^–1^ for the validation set, close to the value presented by the G4 method and surpassing the accuracy of the G3 method of 1.19 kcal mol^–1^ with significantly reduced computational cost. This method shows advantages by eliminating the need for purely empirical corrections, thereby enhancing both efficiency and accuracy in predicting heats of formation.

## 1 Introduction

Many methods in the literature aim to determine thermochemical and spectroscopic properties for chemical systems of different sizes using composite approaches. The composite methods combine lower-level ab initio calculations yielding accurate results with reduced computational efforts compared to higher-level methods.^1−5^

Among the important composite methods are those in the Gaussian-n (Gn) family, developed by Pople, Curtiss, and colaborators.^6−17^ These methods estimate accurate electronic energy by applying linear corrections based on diverse conditions of electronic correlation effects and basis function. Often, in the case of Gn methods, empirical adjustments are employed to rectify inherent shortcomings, like high-level corrections (HLC), harmonic vibrational frequency scaling, atomization energy adjustments, among others.^18−25^ While effective in minimizing deviations from experimental data, it is sometimes unclear if these corrections address deficiencies in electronic correlation effects, basis functions, both, or other overlooked factors, including the quality of experimental data used in properties like enthalpies of formation.

In an attempt to replace some of these corrections with empirical adjustments, the G3S^7^ method was developed. This method is based on the proposals by Gordon and Truhlar^26−30^ for scaling various electronic correlation terms, the PCI-X method by Siegbahn et al.^31^ for parametrized correlation, and the multi-coefficient correlation method (MCCM) by Truhlar et al.^27,30^ With a maximum of six parameters, the G3S method achieved an experimental accuracy level of 0.97 kcal mol^–1^ for calculating 148 enthalpies of formation, using the G2/97^13,32^ experimental reference set.

Despite its effectiveness, G3S has limitations. Its representativeness was constrained by a relatively small structure set (148 molecules), and like many composite methods, it does not incorporate an external validation set to assess the generalizability of the method. This is a common issue, irrespective of whether the method involves adjustable parameters.

These challenges highlight the need for new or improved composite methodologies. The objective of this work is to develop an adaptive composite method by directly adjusting correlation terms and basis functions using neural networks. This approach will utilize four parameters to refine electronic correlation terms and basis function effects to calculate enthalpies of formation. The efficacy and representativeness of the adjustments will be assessed through external validation.

## 2 Computational Methods

It is worth mentioning that the method described here is statistical in nature, employing artificial neural networks. Neural networks and other artificial intelligence methods are designed for classification, optimization, or both. In the first step, the HCA is used to establish a classification criterion based on the absolute error between calculated and experimental enthalpies of formation, grouping molecules into components that minimize the mean absolute error (MAE). Once the optimal number of groups is determined, the second step involves reoptimizing the parameters for each group to enhance the calculated enthalpies of formation for molecules within each group. Finally, the third step employs the ANN for classification and improvement of enthalpy predictions for new systems. With this preliminary outline in mind, the details of these three steps are elaborated in the following sections.

### 2.1 General Adjustments

The molecular internal energy expression is defined by a reference energy (*E*_ref_) modified by electronic correlation (Δ*E*_corr_) and basis function effects (Δ*E*_basis_), along with thermal contribution. Adjustments for atoms and molecules are independent, as shown in the following equations
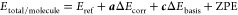
1
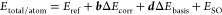
2where ZPE^33^ represents harmonic zero-point energy and *E*_SO_ indicates the spin–orbit correction for the atoms only. The reference energy is computed at the HF/aug-cc-pVDZ level. The electronic correlation correction is given by Δ*E*_corr_ = *E*[QCISD(T)/aug-cc-pVDZ] – *E*[HF/aug-cc-pVDZ], while basis function deficiency is addressed using Dunning type basis functions:^34^ Δ*E*_basis_ = *E*[MP2/aug-cc-pVTZ] – *E*[MP2/aug-cc-pVDZ]. Parameters ***a*** and ***c*** are for molecules, ***b*** and ***d*** are for atoms. Molecular geometries and all thermal effects for all single-point calculations were optimized at B3LYP/6-31G(d) level. Both the basis set increment and geometry optimization steps were performed following a scheme similar to that in the G3MP2(B3) method.^10^ All calculations were performed using 5d and 7f functions, which define the angular momentum components of d- and f-orbitals in standard basis sets, for basis set consistency with modern electronic structure codes. The use of 5d functions avoids the inclusion of an additional unphysical s orbital that can arise from 6d functions, mitigating potential basis set linear dependency issues. Preliminary tests showed negligible differences in the results compared to calculations using 6d functions, ensuring that the conclusions drawn remain valid. Thermal corrections were included only in the enthalpy of formation calculation, determined using a well-established method in the literature.^35,36^ The Gaussian 09 package^37^ was used for all calculations.

Parameters ***a***, ***b***, ***c***, and ***d*** were initially optimized for each molecule against G3/05 test set enthalpies of formation,^16^ aiming to minimize errors between calculated and experimental enthalpies of formation. Starting with an initial value of one for each parameter, the BFGS gradient method^38^ was used for optimization. The optimal parameters were arranged in a 248 × 4 matrix, containing the four values for each of the 248 molecules. Each column of the matrix was centered on its respective average value. Subsequently, a hierarchical clustering analysis using the Ward method^39^ was applied to generate up to four different groups. The clustering criterion was based on the similarity of the values of the four adjusted parameters.

Following the characterization of each cluster through Hierarchical Cluster Analysis (HCA), 10% of the molecules from each cluster were set aside for validation of the final model. The remaining 90% were used to optimize the four parameters that represent the optimal settings for each respective group. Considering the available sample size of molecules, up to four different groups were formed during the clustering process. This approach helped to avoid parameter overfitting and ensured the representativeness of each group. As a result, four groups labeled G1, G2, G3, and G4 were established, containing 36, 94, 38, and 80 molecules, respectively.

**Figure 1:**
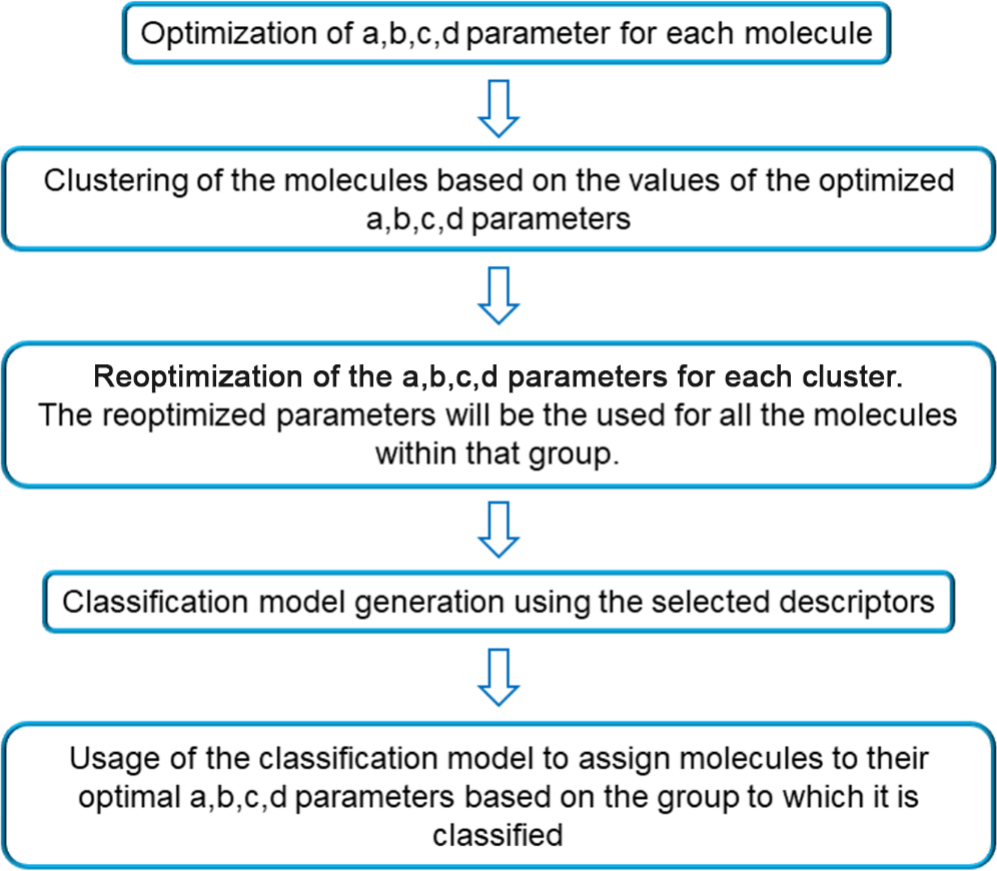
Workflow of the ANN classification model, including parameter optimization, clustering, reoptimization, and classification.

Once the training and validation sets were selected and the respective parameters for each training set were optimized, a classification model was constructed. This model assigns unknown molecules to one of the four groups to determine the optimal set of parameters (***a***, ***b***, ***c***, ***d***) for use in eqs 1 and 2. The steps of this general method are summarized in Figure 1.

### 2.2 Classification Models

To train a classification model for assigning unknown molecules to one of the generated groups, a set of features was designed based on the information available from the calculations necessary for implementing eqs 1 and 2. Table S1 in the Supporting Information lists the features used as input for training the model. Three classification methods were tested: K-nearest neighbors (KNN),^40^ support vector machine (SVM),^41^ and artificial neural networks (ANN).^42^ Each method was employed to develop the best classification model through a semiexhaustive search for optimal model parameters.

For SVM models, the regularization parameter *C*, which controls the trade-off between model complexity and classification accuracy, and the kernel coefficient γ, which defines the influence range of a training example, were scanned across linear, radial, and polynomial kernels. The *C* and γ parameters were scanned in the range of 0.1–100 and 0.01–10, respectively. In the KNN model, the sole adjusted parameter was the number of nearest neighbors. Here, schemes with 3, 5, and 7 neighbors were experimented. For the ANN model, given the large number of parameters such as the number of layers, neurons, regularizers, etc., a manual search for the best set of parameters was carried out.

**Figure 2:**
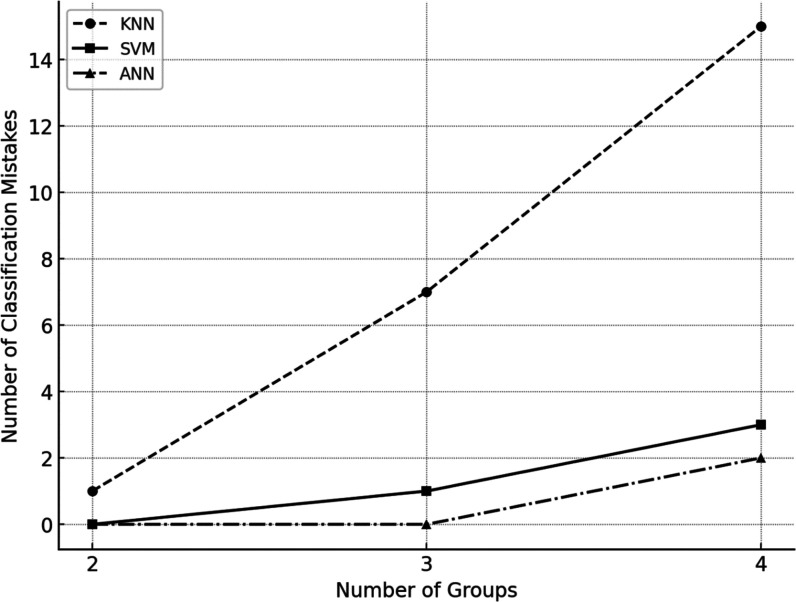
Comparison of the different classification methods with respect to the number of mistakes made by each model as the number of possible groups increases.

Preliminary tests indicated that as the complexity of the classification problem increased with the number of groups considered, the ANN classification showed superior performance (Figure 2). The optimized ANN model contained 2 hidden layers, each using the Rectified Linear Unit (ReLU) activation function and containing 8 fully connected neurons plus 1 bias corrector. The output layer contained 4 units, each representing one of the groups considered in the classification. A key feature of ANN classification is its use of a softmax distribution in the output. This allows for the application of a weighted average of the adjusted parameters ***a***, ***b***, ***c***, and ***d*** from each group, based on the respective probabilities of a molecule belonging to each group, rather than using the parameters of the most likely one.

## 3 Results and Discussion

### 3.1 ANN-Driven Predictions

Different ANN classification models were created, categorizing molecules into 2, 3, and 4 groups. Table 1 details the mean absolute error (MAE) results for the enthalpies of formation of test set molecules across these three levels of grouping. These results were obtained using two approaches: the most probable group and the weighted average according to the softmax distribution. The MAEs for both the test set and the entire G3/05 set predominantly ranged from 0.8 to 2.0 kcal mol^–1^. The uniformity of MAE values across all groups suggests a degree of consistency in the calculated errors.

**Table 1:** Mean Absolute Errors (MAE in kcal mol^–1^) in Calculated Enthalpies of Formation Compared to Experimental Data: (a) for the Test Set, and (b) for All Molecules in the G3/05 Group

	test set		complete G3/05	
number of groups	Max.Prob.	average	Max.Prob.	average
1	2.15	2.15	2.15	2.15
2	1.90	1.73	1.99	1.95
3	1.27	1.17	1.31	1.28
4	1.17	0.89	1.26	1.12

Analysis of the data in Table 1 enabled the identification of the most effective grouping method for the G3/05 set, a critical step in finalizing the proposed configuration method. A trend of improved accuracy in adjustments was observed with an increased number of optimization groups. However, this improvement trend is not immediately apparent since more groups or classes for the molecules also complicate the classification process, potentially increasing the mean absolute error. To avoid diminishing the generalizability of the method, the number of groups was not excessively increased due to the limited size of the available molecule set.

Additionally, when increasing the number of groups beyond 4, some groups contained too few molecules, leading to increasingly unreliable optimized parameters for those groups. Moreover, no significant improvement in the mean absolute error (MAE) was observed for either the training or validation sets to justify the inclusion of additional groups. For demonstration purposes, the MAE values for the validation set with 4, 5, and 6 groups are as follows: 4 groups: 0.89 kcal mol^–1^, 5 groups: 0.85 kcal mol^–1^, and 6 groups: 0.84 kcal mol^–1^. It is worth noting that for these tests, the same ANN model structure was applied in terms of the number of hidden layers and type of activation functions, with the only modification being the number of output units to match the number of groups. The model was not further optimized for 5 or 6 groups, as it was for the 4-group model.

**Figure 3:**
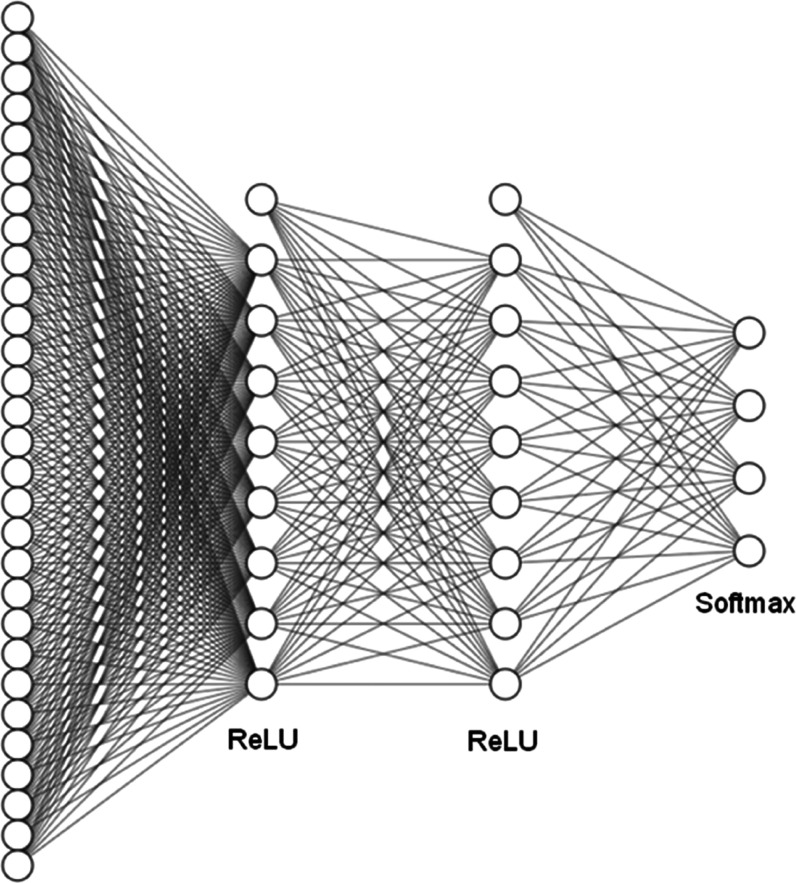
Framework of the ANN classification model. ReLU functions were used in the hidden layers, and softmax functions were used in the output layer.

Figure 3 illustrates the optimized neural network configuration that presented the best performance. This network, tasked with selecting the parameters ***a***, ***b***, ***c***, and ***d*** for each molecule, demonstrated *R*^2^ values of 0.90 and 0.89 for the training and test sets, respectively. Table 2 presents the calculated enthalpies of formation for the G3/05 set molecules, alongside deviations from experimental values.

**Table 2:** Experimental (Exp.) and Calculated (Calc.) Enthalpies of Formation (in kcal mol^–1^)^a^

molecule	Exp.	Cal.	molecule	Exp.	Calc.
SiF_4_	–386.0	0.35	CH_2_CO	–11.4	–0.67
PF_5_	–381.1	2.42	CH_3_CH_2_NH_2_	–11.3	0.51
C_2_F_6_	–321.3	2.59	cycC_5_H_10_NH	–11.3	–0.21
Al_2_Cl_6_	–309.7	0.44	C_2_H_5_SH-ethanethiol	–11.1	0.35
SF_6_	–291.7	2.27	NH_3_	–11	–0.45
AlF_3_	–289.0	–0.65	CBrCl_3_	–10	–1.89
BF_3_	–271.4	2.05	CH_3_SCH_3_	–8.9	0.58
PF_3_	–229.1	0.11	C_4_H_4_O	–8.3	1.89
C_6_F_6_	–228.4	2.56	C_4_H_8_S	–8.2	–1.31
CF_4_	–223.0	1.98	CH_3_SiH_3_	–7	0.50
C_6_F_5_Cl	–194.1	–2.09	(CH_3_)_3_N	–5.7	0.56
BeF_2_	–190.3	–0.03	CH_3_SH	–5.5	0.79
CF_3_Cl	–169.5	2.08	CH_3_NH_2_	–5.5	–0.62
CHF_3_	–166.6	1.01	ClFO_3_	–5.1	0.08
SiCl_4_	–158.0	–2.15	H_2_S	–4.9	0.64
C_2_F_4_	–157.4	3.69	(CH_3_)_2_NH	–4.4	–0.43
CF_3_Br	–155.0	1.63	SCl_2_	–4.2	–0.98
COF_2_	–149.1	–4.23	H_2_COH	–4.1	0.19
AlCl_3_	–139.7	–0.36	C_4_H_8_,isobutane	–4.0	–0.04
CH_3_–C=OOC=OCH_3_	–136.8	0.85	S_2_Cl_2_	–4.0	–0.16
POCl_3_	–133.8	9.95	CH_3_CH_2_O	–3.7	1.00
CF_3_CN	–118.4	–1.58	C_4_H_6_O	–3.3	–0.98
CH_3_C=OOCH(CH_3_)_2_, isopropylacetate	–115.1	–0.25	C_2_Cl_4_	–3.0	0.37
CF_3_	–111.3	1.25	CH_3_CO (2A)	–2.4	–0.22
CH_2_F_2_	–107.7	0.23	C_4_H_8_NH	–0.8	0.28
CH_3_COF	–105.7	1.00	F_2_	0.0	0.80
CH_3_COOH	–103.4	0.40	H_2_	0.0	0.71
CHF_2_Br	–101.6	0.12	Cl_2_	0.0	0.50
CH_3_COOCH_3_	–98.4	0.28	O_2_	0.0	0.31
BCl_3_	–96.3	–1.01	N_2_	0.0	–0.35
SO_3_	–94.6	–0.17	SO	1.2	–0.80
CO_2_	–94.1	0.70	PH_3_	1.3	–0.38
MgCl_2_	–93.8	–0.33	N_2_O_4_	2.2	–0.31
CH_3_–CH(OCH_3_)_2_	–93.1	2.63	ClNO_2_	2.9	0.80
HCOOH	–90.5	1.37	CH_3_O	4.1	–0.01
(CH_3_)_2_SO_2_	–89.7	–1.36	CH_3_–CH=CH_2_	4.8	0.11
PCl_5_	–86.1	1.53	(CH_3_)_2_CHCN	5.6	–0.66
HCOOCH_3_	–85.0	2.48	F_2_O	5.9	2.10
SO_2_Cl_2_	–84.8	6.63	C_4_H_8_,cyclobutane	6.8	0.77
LiF	–80.1	–1.18	NO_2_	7.9	–0.45
(CH_3_)_2_CHOCH(CH_3_)_2_	–76.3	–0.02	SiH_4_	8.2	1.16
C_4_H_8_O_2_,(*para*)	–75.5	1.40	CH_2_=CHCl	8.9	1.75
(CH_3_)_3_COH	–74.7	0.26	OH	9.4	0.99
C_6_H_4_F_2_, 1,3-difluorobenzene	–73.9	–1.40	HCO	10.0	–1.02
C_6_H_4_F_2_, 1,4-difluorobenzene	–73.3	–1.43	C_6_H_5_–CH_3_,toluene	12.0	–1.01
SO_2_	–71.0	0.67	(CH_3_)_3_C	12.3	–0.08
NaF	–69.4	–2.11	C_6_H_5_Cl	12.4	3.12
PCl_3_	–69.0	–1.85	ClNO	12.4	–0.15
(CH_3_)_3_COCH_3_	–67.8	0.38	C_2_H_4_	12.5	–0.06
(CH_3_)_2_CHOH	–65.2	0.76	C_3_H_6_,cyclopropane	12.7	–0.24
HF	–65.1	0.71	P_4_	14.1	–1.33
AlF	–63.5	0.25	CH_3_C=OCCH	15.6	–0.96
CH_3_CH_2_COCH_2_CH_3_	–61.6	–0.85	C_5_H_8_,isoprene	18.0	–0.46
CH_3_CH_2_OCH_2_CH_3_	–60.3	0.54	CH_3_CN	18.0	0.56
CH_3_COCl	–58.0	–0.49	C_2_H_3_Br	18.9	–1.55
H_2_O	–57.8	0.11	Si_2_H_6_	19.1	1.79
CH_3_COCH_2_CH_3_, methylethylketone	–57.1	–0.47	N_2_O	19.6	–0.53
CH_3_CONH_2_	–57.0	0.08	C_2_H_4_S	19.6	0.41
CH_3_CH_2_OH	–56.2	1.04	C_6_H_6_	19.7	–1.13
Si(CH_3_)_4_	–55.7	–3.97	N_2_O_3_	19.8	–0.16
C_5_H_10_O	–53.4	–1.11	C_5_H_6_S,methylthiophene,	20.0	–2.59
Cl_2_CO	–52.4	–0.73	C_4_H_6_S	20.8	–0.79
CH_3_COCH_3_	–51.9	–0.34	C_6_H_5_NH_2_	20.8	–1.35
C_2_H_5_OCH_3_methyl–ethyl-ether	–51.7	1.50	(CH_3_)_2_CH	21.5	0.76
(CH_3_)_2_CHCHO,isobutanal	–51.6	–1.82	NO	21.6	–0.91
HCOCOH	–50.7	2.48	H_2_NNH_2_	22.8	–0.90
C_8_H_18_	–49.9	c1.45	ClO	24.2	–0.91
H_3_COH	–48.0	1.51	C_5_H_7_N	24.6	–0.91
C_5_H_8_O	–45.9	–1.47	C_6_H_8_,1,4-cyclohexadiene	25.0	–0.80
C_7_H_16_	–44.9	1.30	C_6_H_5_Br	25.2	–2.97
CH_3_OCH_3_	–44.0	1.52	C_6_H_8_,1,3-cyclohexadiene	25.4	–0.44
C_4_H_8_O (tetrahydrofuran)	–44.0	–0.76	C_4_H_5_N	25.9	–1.07
NaCl	–43.8	–1.58	CH_2_CHCHCH_2_,butadiene	26.3	–0.59
(CH_3_)_3_CCl	–43.5	1.38	C_4_H_4_S	27.5	–1.52
C_6_H_14_,methylpentane	–41.1	0.54	CS_2_	28.0	–1.09
SiCl_2_	–40.3	–2.43	C_2_H_5_	28.9	0.67
C_5_H_12_,neopentane	–40.2	–0.08	CH_3_S	29.8	0.96
C_6_H_14_	–39.9	1.18	C_2_H_4_NH	30.2	–0.10
CH_3_CHO	–39.7	0.23	S_2_	30.7	–1.30
CH_3_CH_2_CH(CH_3_)NO_2_, nitro-*s*-butane	–39.1	0.86	HCN	31.5	0.43
ClF_3_	–38.0	–19.68	ClCN	32.9	–0.83
CH_3_CH_2_CH_2_CH_2_Cl	–37.0	0.72	PH_2_	33.1	1.00
(CH_3_)_2_SO	–36.2	0.08	LiH	33.3	0.37
C_6_H_13_Br	–35.4	–1.33	C_5_H_5_N	33.6	–0.51
C_5_H_12_,*n*-pentane	–35.1	0.77	Na_2_	34.0	0.26
CH_2_=CHF	–33.2	0.40	O_3_	34.1	0.92
OCS	–33.0	0.64	HS	34.2	0.45
H_2_O_2_	–32.5	2.01	P_2_	34.3	–1.02
C_4_H_10_,ISO	–32.1	0.37	C_4_H_6_,2-butyne	34.8	–0.18
NF_3_	–31.6	0.02	CH_3_	35.0	0.35
CH_3_CH_2_CH_2_Cl	–31.5	1.99	C_10_H_8_,naphthalene	35.9	–0.89
C_4_H_10_,trans	–30.0	0.75	C_4_H_6_,cyclobutene	37.4	–0.92
C_6_H_12_	–29.5	0.38	CH_3_–CH=C=CH_2_	38.8	0.36
C_6_H_4_O_2_	–29.4	–3.49	CH_2_CHCN	43.2	–1.47
(CH_3_)_3_CNH_2_,*t*-butylamine	–28.9	0.13	LiNa	43.4	0.33
C_6_H_5_F	–27.7	4.53	CH_3_CCH	44.2	0.27
COBr_2_	–27.1	–3.29	C_5_H_8_,spiropentane	44.3	0.02
C_2_H_5_Cl	–26.8	1.43	NH_2_	45.1	1.43
CO	–26.4	–0.47	CH_2_=C=CH_2_, (allene)	45.5	0.50
(CH_3_)_3_CSH	–26.2	0.49	C_4_H_4_N_2_,1,4-dipyridine	46.8	–3.08
H_2_CO	–26.0	0.46	C_4_H_4_N_2_,pyrimidine	46.9	1.48
C_3_H_8_,propene	–25.0	0.56	SiH_3_	47.9	0.99
CHCl_3_	–24.7	–0.94	C_4_H_6_-methylenecyclopropane	47.9	1.90
SiO	–24.6	–1.20	NCCH_2_CH_2_CN	50.1	0.68
CH_3_CH=CHCHO	–24.0	–0.39	Li_2_	51.6	0.27
C_3_H_7_Br	–23.8	–2.35	C_4_H_6_,bicyclo	51.9	–1.67
C_6_H_5_OH	–23.0	–1.01	C_2_H_2_	54.2	–0.28
CCl_4_	–22.9	–1.06	SiH_2_,singlete	65.2	1.33
CH_2_Cl_2_	–22.8	–0.58	C_3_H_4_	66.2	–1.30
HCl	–22.1	0.80	CS	66.9	–0.69
C_2_H_6_	–20.1	0.38	C_10_H_8_,azulene	69.1	–2.92
CH_3_Cl	–19.5	–0.13	C_8_H_8_	70.7	–1.18
C_5_H_10_,cyclopentane	–18.3	0.17	C_2_H_3_	71.6	0.40
CH_3_CH_2_SSCH_2_CH_3_	–17.9	–1.21	NCCN	73.3	–0.46
CH_4_	–17.9	0.03	C_6_H_5_, phenil_radical	81.2	–2.87
CH_3_NO_2_	–17.8	–1.10	BeH	81.7	–0.42
HOCl	–17.8	1.13	NH	85.2	0.77
C_3_H_6_Br_2_	–17.1	–0.24	SiH_2_,triplet	86.2	1.00
CH_3_ONO	–15.9	0.30	CH_2_,triplet	93.7	–0.15
C_5_H_10_S	–15.2	–1.55	CH_2_,singlet	102.8	0.46
C_2_H_5_Br	–14.8	–0.94	CN	104.9	–2.21
ClF	–13.2	0.39	CCH	135.1	–1.14
C_5_H_8_Br_2_	–13.1	–0.32	Si_2_	139.9	–1.15
C_2_H_4_O	–12.6	0.94	CH	142.5	0.43

a The Calc. column refers to the mean absolute error (MAE) between the calculated and the experimental values.

### 3.2 Group Formation and Adjustments

The rationale behind the adaptive composite method lies in creating a calculation approach that is (a) versatile across various molecular systems without significantly compromising accuracy; (b) representative, having undergone validation against an external set of test molecules; (c) capable of providing a basic level of physical rationale in its corrections; and (d) computationally efficient.

The rigor and versatility of the method are highlighted in Table 1, which shows a 20% reduction in the mean error for the test set when using two adjustment possibilities for the ***a***, ***b***, ***c***, and ***d*** parameters in enthalpy of formation calculations. Expanding to four adjustment possibilities leads to a 46% reduction in MAE. Notably, increased adjustment options introduce complexity, potentially complicating the accurate classification of molecules and affecting enthalpy of formation calculations. This underscores the importance of a robust classification algorithm.

An attempt to understand how groups are formed in relation to the chemical composition of the molecules can be insightful. Table 3 presents the elemental composition of groups 1 and 2. The division of the G3/05 set into two groups, as analyzed in Table 3, reflects the chemical composition of the molecules in each group. This information aids in identifying where the effects of electronic correlation and basis function enhancement, implemented in the method, are either under or overestimated.

**Table 3:** Normalized Rate of Occurrence of Each Element in Each Group^a^

atoms	Group1	Group 2
H	0.50	4.57
C	0.50	2.58
N	0.03	0.23
O	0.19	0.48
F	1.14	0.25
Si	0.06	0.06
P	0.00	0.06
S	0.03	0.14
Cl	0.61	0.26
Br	0.14	0.04

a The count was made over the complete G3/05 set.

Comparing the parameters of each group (Table 4), it is noted that the differences between scaling parameters ***a*** and ***b***, associated with the electronic correlation term, and ***c*** and ***d***, associated with the basis set correction term are very small for Group 1. However, both differences (***a***_**molecule**_ – ***b***_**atoms**_) and (***c*****_molecule_** – ***d*****_atoms_**) are large for Group 2. Table 3 shows that Group 1 contains most molecules with fluorine, chlorine, and bromine. The presence of these electron-rich elements justifies the need for approximately equal scaling of the correlation correction term for atoms and the molecules containing these elements in Group 1. On the other hand, Group 2 has a higher presence of nitrogen, carbon, hydrogen, and oxygen. These elements are associated with the larger organic molecules contained in Group 2, which require a higher scaling of the basis set correction term in the molecule due to the greater spatial span of their electronic density. Moreover, the greater value of ***b***_**atoms**_ when compared to ***a***_**molecule**_ in Group 2 seems to counter the overestimation of the correlation correction term for the molecules in this group.

**Table 4:** Optimal Parameters Obtained for Groups 1 and 2 in the Two-Group Clustering Stage

parameters	Group 1	Group 2
***a***	0.962702	0.971345
***b***	0.970629	1.015386
***c***	1.391322	1.420572
***d***	1.399096	1.348204

Moving to the grouping in 3 groups (Table 5), a separation of the organic molecules in Group 2 is observed, with two further subgroups: oxygenated and halogenated molecules, and nitrogenated molecules with almost no halogens. Further subgrouping into 4 groups results in the division of Group 3 into molecules richer in oxygen, nitrogen, and sulfur from the smaller molecules containing fluorine and chlorine. Similarly to what was observed in the first grouping (Table 6). Similarly to the grouping in 2 groups, the difference (***a***_**molecule**_ – ***b***_**atoms**_) is smaller for the groups containing more halogens (Groups 1 and 3) and higher for groups without these elements. Additionally, the difference (***c***_**molecule**_ – ***d***_**atoms**_) is higher in the groups containing molecules with longer carbon chains (Groups 2 and 4). Further subgrouping would likely increase chemical specificity within each group but also raise the risk of overfitting. A complete breakdown of the molecules present in each of the four final groups is presented (Table S2) in the Supporting Information along with all the optimized parameters for each group (Table S3).

**Table 5:** Occurrence Rate of Each Element in the Molecules Present in the Generated Groups

atoms	Group 1	Group 2	Group 3
H	0.50	5.95	3.47
C	0.50	3.56	1.79
N	0.03	0.33	0.14
O	0.19	0.22	0.69
F	1.14	0.05	0.42
Si	0.06	0.02	0.08
P	0.00	0.09	0.04
S	0.03	0.07	0.19
Cl	0.61	0.09	0.40
Br	0.14	0.03	0.05

**Table 6:** Occurrence Rate of Each Element in the Molecules Present in the Generated 4 Final Groups

atom	Group 1	Group 2	Group 3	Group 4
H	0.50	5.95	0.87	4.70
C	0.50	3.56	0.66	2.33
N	0.03	0.33	0.24	0.10
O	0.19	0.22	0.58	0.74
F	1.14	0.05	0.82	0.22
Si	0.06	0.02	0.11	0.08
P	0.00	0.09	0.03	0.05
S	0.03	0.07	0.13	0.22
Cl	0.61	0.09	1.00	0.11
Br	0.14	0.03	0.05	0.05

Hierarchical clustering analysis reveals that groupings of more than two categories emerge as branches of the initial grouping. This branching provides insights into the chemical specificity of each group and its constituent molecules, as well as the necessary corrections to be applied when calculating the properties of these groups (Table 7).

**Table 7:** Optimal Parameters Obtained for Groups 1, 2, and 3 in the Three-Group Clustering Stage

parameters	Group 1	Group 2	Group 3
***a***	0.962702	0.859644	0.999648
***b***	0.970629	0.981000	1.028248
***c***	1.391322	1.362546	1.300952
***d***	1.399096	1.237292	1.265845

### 3.3 General Implementation

This section outlines the process for determining the ***a***, ***b***, ***c***, and ***d*** parameters for a given molecule. As an example, we will calculate these parameters for **SiF**_**4**_. The method involves several steps, which are detailed below:

#### 3.3.1 Generation of the Feature Array

The feature array is constructed using the descriptors listed in Table S1. For **SiF**_**4**_, the feature array is defined as **SiF_4_**: features = [0.0, 0.0, 0.0, 0.0, 0.0, 0.0, 0.0, 4.0, 0.0, 0.0, 0.0, 1.0, 0.0, 0.0, 0.0, 0.0, −0.93, 0.38, 1.31, −0.39, 0.49, −0.44, 0.87, 0.01, −0.0, 0.07, 0.81, 180.48, 4.96]

#### 3.3.2 Normalization of the Feature Array

The feature array is normalized using the mean and standard deviation values obtained from the training step (provided in the Supporting Information). The normalized feature array is calculated as normalized = (features – mean)/std.

#### 3.3.3 Feeding the Normalized Array into the ANN Model

The normalized feature array is then input into the pretrained Artificial Neural Network (ANN) model. The output is a probability distribution representing the likelihood of the molecule belonging to each of the four predefined groups: Prob. = softmax(ReLU(ReLU([normalized]·[*W*1])·[*W*2] + *b*2)·[*W*3] + *b*3), where softmax(*x*_*i*_) = e^*x*_i_–max^(*x*)/(∑_*j* = 1_^*n*^ e^*x_j_*–max^(*x*)) and ReLU(*x*_*i*_) = max(0, *x*_*i*_).

#### 3.3.4 Calculation of the Final Parameters

The final ***a***, ***b***, ***c***, and ***d*** parameters are computed as the weighted average of the parameters listed in Table S3, based on the probability distribution calculated in the previous step. For **SiF**_**4**_, the calculation is as follows
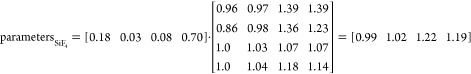
3**Note**: For clarity, the matrix values have been truncated. For practical calculations, all significant figures should be used to ensure accuracy.

A plug-and-play implementation of the present method is accessible through the web application available at: https://gabrielcesar9.github.io/ANNG3S/. Users can input energy values from single point calculations to obtain the optimal parameters ***a***, ***b***, ***c***, and ***d***, as well as the final energy, atomization energy, and heat of formation for their system. Additionally, the parameters ***a***, ***b***, ***c***, and ***d*** can be calculated using the ANN mathematical model, which involves the dot product of the matrices containing the weight parameters of the model (eq 3) as implemented in a simple python function made available through the script composite.py in the Supporting Information.

## 4 Conclusions

This study highlights the increasing importance of machine learning algorithms in determining chemical properties and developing high-accuracy, cost-effective computational methods. An adaptive composite method was devised, utilizing advancements in computational chemistry to effectively balance accuracy, representativeness (externally validated), and processing efficiency.

The validity of the method is demonstrated by its ability to approximate enthalpies of formation with an accuracy of about 0.89 kcal mol^–1^ for molecules in the validation set, i.e., those outside the training set. This reflects the robustness of the model when applied to unseen data. Additionally, the method offers computational efficiency comparable to the most accurate Gn methods. It provides a slight improvement over G3 with respect to experimental data and approaches the performance of the G4 method, while reducing computational costs by utilizing smaller basis sets and lower-order perturbation computations.

To quantify this, the mean absolute errors (MAE) compared to experimental values for some Gaussian-n methods and the proposed ANN-G3S method on the training set are as follows: G3:1.19 kcal mol^–1^, G3(MP2): 1.40 kcal mol^–1^, G4:0.80 kcal mol^–1^, G4(MP2): 0.99 kcal mol^–1^, and ANN-G3S: 1.11 kcal mol^–1^. These results demonstrate that the ANN-G3S method performs slightly better than G3 and approaches G4′s accuracy, while maintaining greater computational efficiency.

A key feature of this method is the transparent implementation of empirical adjustments in electronic correlation terms and basis functions, which make clear the limitations of the theoretical levels used. This insight lays the groundwork for future optimizations, aimed at more precisely modeling specific system types, informed by identified deficiencies in parameters ***a***, ***b***, ***c***, and ***d*** concerning modeling correlation and basis set effects.

Future efforts will focus on refining this method, particularly its core formulation (eq 1) and the classification process crucial for deriving optimal parameters with maximum confidence. These attempts will aim to enhance the adaptability and predictive power of the method, further solidifying its role in electronic calculations.

## Supporting Information Available

All tools and data necessary to ensure reproducibility and ease of access; data vector used as the information source for model training (Table S1); parameter values of each of the four final groups (Table S2); parameter values of each of the four final groups (Table S3); additionally, a Python script (composite.py) is provided to enable users to calculate molecular properties and classify molecules locally, without relying on external web servers, this program offers a straightforward implementation of the proposed methodology (PDF)
