# Supplementary material for: A Preliminary Neural Network-Based Composite Method for Accurate Prediction of Enthalpies of Formation
Source: J Chem Theory Comput. 2024 Dec 12;20(24):10922–30. doi: 10.1021/acs.jctc.4c01351 (PMC11672661; doi:10.1021/acs.jctc.4c01351)
Supplement: Supplementary file 1 — ct4c01351_si_001.pdf [file ct4c01351_si_001.pdf]

SUPPLEMENTARY MATERIAL

A Preliminary Neural Network-Based Composite Method for Accurate Prediction of Enthalpies of Formation

Gabriel César Pereira e Rogério Custodio[*]

Instituto de Química

Universidade Estadual de Campinas

Barão Geraldo

13083-970 Campinas – São Paulo, Brazil, P. O. Box 6154

[*] Corresponding author: e-mail: rogerct@unicamp.br, Tel.: +55-19-35213104; fax: +55-19-35213023.

Index

SUPPLEMENTARY MATERIAL....................1

Table S.1. Data vector used as the information source for model training....................3

Table S.2. Molecules present in each of the four final groups. 4

Table S.3. Parameter values of each of the four final groups. 8

Python Script (composite.py) 9

Table S.1. Data vector used as the information source for model training.

Num. of H	$QCISD(T)/pvDz_{molecule} - HF/pvDz_{molecule}$
Num. of Li	$QCISD(T)/pvDz_{molecule} - Em^{*}$
Num. of Be	$HF/pvDz_{molecule} - Em^{*}$
Num. of B	$MP2/pvDz_{pvTz} - MP2/pvDz_{pvDz}$
Num. of C	$Ea^{*} - QCISD(T)/pvDz_{molecule}$
Num. of N	$Ea^{*} - HF/pvDz_{molecule}$
Num. of O	$Ea^{*} - Em$
Num. of F	$Thermal_{corr}{}^{*} - ZPE$
Num. of Na	$\sum SO_{atom}{}^{*}$
Num. of Mg	$\sum Large_{atom} - Large_{molecule}{}^{*}$
Num. of Al	$\sum QCISD(T)/pvDz_{atom} - QCISD(T)/pvDz_{mo}$[illegible]
Num. of Si	$\sum \Delta_f H^o(0K)_{atom}{}^{*}$
Num. of P	$\sum \Delta_f H^o(298K)_{atom} - \Delta_f H^o(0K)_{atom}{}^{*}$
Num. of S	
Num. of Cl	
Num. of Br	

$*$ Em refers to the molecular energy calculated thourgh Eq. 1 with all the four parameters set to 1.
$*$ Ea refers to the total atomic energy calculated thourgh Eq. 2 with all the four parameters set to 1.
$*$ $Thermal_{corr}$ refers to the thermal correction to the enthalpy.
$*$ SO_{atom} refers to the spin-splitting correction for each atom.
* $Large$ refers to the difference MP2(aug-cc-pvTz) - MP2(aug-cc-pvDz)
*$\Delta_f H^o(0K)_{atom}$ refers to the atomic heat of formation at 0K.

*$\sum \Delta_f H^o(298K)_{atom} - \Delta_f H^o(0K)_{atom}$ refers to the atomic enthalpy correction.

Table S.2. Molecules present in each of the four final groups.

Group 1	Group 2	Group 3	Group 4
LiF	SO3	AlCl3	HCOOCH3
CF3Cl	SO2Cl2	SiCl4	PF5
CHF3	POCl3	AlF3	PF3
CF3Br	(CH3)2SO2	SF6	(CH3)3COH
CH2F2	SO2	C6F5Cl	CH3COOCH3
C2F6	C6H13Br	BeF2	(CH3)3COCH3
MgCl2	Si(CH3)4	BCl3	PCl3
C2F4	C7H16	BF3	C4H8O2,(para)
CF4	CH3CONH2	CF3CN	HCOOH
CF3	ClF3	COF2	CH3-C=OOC=OCH3
CHF2Br	C8H18	NaF	SiF4
HF	C5H12,neopentane	C6F6	C6H4F2,1,3-DiFluorobenzene,
AlF	(CH3)2SO	PCl5	C6H4F2,1,4-DiFluorobenzene,
OH	C5H12,n-pentane	SiCl2	CH3-CH(OCH3)2
CCl4	C6H14,methylpentane	Cl2CO	(CH3)2CHOCH(CH3)2
SiH2,singlet	C6H14	H2O	CO2
COBr2	C6H12	NaCl	CH3COF
CHCl3	C6H5F	H2O2	CH3C=OOCH(CH3)2,isopropylacetate
F2O	(CH3)3CNH2,t-butylamine	'Al2Cl6	CH3COOH
NF3	C3H7Br	CH2Cl2	HCOCOH
HOCl	C6H4O2	S2Cl2	H3COH
Li2	C6H5OH	SCl2	CH3COCH2CH3,methylethylketone
LiH	CH3NH2	H2S	C2H5OCH3,methyl-ethyl-ether
H2	C3H8,propene	SiH4	CH3CH2COCH2CH3
HCl	C10H8, naphthalene	ClO	CH3CHO
Na2	C4H5N	NO2	(CH3)2CHCHO,isobutanal
SiH2,triplet	C4H4N2,1,4-dipyridine	SiH3	CH3COCH3

Cl2	C6H5NH2	CH2=CHCl	C5H10O
ClF	C4H10,trans	N2O4	CH3COCl
HS	C4H10,ISO	CH3Cl	CH3OCH3
LiNa	C6H5Cl	N2O3	CH3CH2CH(CH3)NO2,nitro-s-butane
C2Cl4	C8H8	CH2,singlet	CH3CH2OCH2CH3
O3	C5H6S,methylthiophene,	ClNO	C4H8O (tetrahydrofuran)
CBrCl3	C5H5N	C3H6Br2	(CH3)3CCl
CH	HCN	O2	(CH3)2CHOH
F2	C4H4O	CH3ONO	C5H8O
	CH3SiH3	BeH	CH2=CHF
	CH3CH2NH2	ClNO2	CH3CH2OH
	CH4		CH3CH2CH2CH2Cl
	CH2CHCN		CH3CH2CH2Cl
	C4H6,cyclobutene		CO
	C4H6O		H2CO
	C4H8,cyclobutane		N2O
	C6H5,phenil_radical		C2H5Cl
	C2H4		CH2CO
	ClFO3		OCS
	PH3		CH3CH=CHCHO
	CH3-CH=CH2		SiO
	NH3		C2H4S
	C5H10,cyclopentane		(CH3)3CSH
	C3H6,cyclopropane		CH3CH2SSCH2CH3
	(CH3)3N		CH3CO (2A)
	C2H2		CH3O
	CH2CHCHCH2,butadiene		CH3SCH3
	C6H5-CH3,toluene		C4H6S

	cycC5H10NH		C2H3Br
	C2H4NH		CS2
	C2H6		CH2,triplet
	P2		C5H10S
	(CH3)2NH		NH
	CN		HCO
	C4H4S		CH3S
	C6H8,1,3-cyclohexadiene		CS
	P4		Si2H6
	C5H7N		H2COH
	C4H6,bicyclo		PH2
	C4H6,2-butyne		CH3CH2O
	CH3CCH		NO
	CH3CN		CH3NO2
	CH3		ClCN
	C4H8NH		C2H5SH,ethanethiol
	(CH3)2CH		Si2
	NCCH2CH2CN		NH2
	C6H6		C5H8Br2
	(CH3)2CHCN		SO
	CCH		C2H5Br
	C4H4N2,pyrimidine		C4H8S
	CH3-CH=C=CH2		S2
	C4H8,isobutane		C2H4O
	C5H8,isoprene		CH3SH
	C3H4		
	(CH3)3C		

	C2H5		
	CH2=C=CH2,(allene)		
	CH3C=OCCH		
	C10H8,azulene		
	C6H8,1,4-cyclohexadiene		
	H2NNH2		
	C6H5Br		
	N2		
	C4H6-methylenecyclopropane		
	C2H3		
	C5H8,spiropentane		
	NCCN		

Table S.3. Parameter values of each of the four final groups.

	a	b	c	d
**Group 1**	0.962702	0.970629	1.391322	1.399096
**Group 2**	0.859644	0.981000	1.362546	1.237292
**Group 3**	1.002802	1.030844	1.079071	1.076161
**Group 4**	1.000749	1.041345	1.180963	1.141014

Python Script (composite.py)

The script is compatible with any version of Python and requires only the pre-installation of the scientific Python package NumPy. At the beginning of the script, the necessary input parameters for calculating a, b, c, and d, as well as the heat of formation, are clearly defined. These inputs include: the number of atoms for each representative element, HF, QCISD(T), MP2(aug-cc-pVDZ), and MP2(aug-cc-pVTZ) energies, along with Zero-Point Energy (ZPE) and thermal corrections. As an example, the final line of the script demonstrates its use by calculating the parameters for the SiF_4 molecule.

```
def cal_parameters(H,Li, Be, B, C, N, O, F, Na, Mg, Al, Si, P, S, Cl, Br, HF, QCISDT, MP2dz_m, MP2tz_m, ZPE, CorrT):
    import numpy as np

    COUNT_A = np.array([H,Li, Be, B, C, N, O, F, Na, Mg, Al, Si, P, S, Cl, Br,])

    Ahf = np.array([-0.4993343, -7.4324257, -14.5723798, -24.5305738, -37.6877633, -54.3931834, -74.7966008, -99.3810918, -161.8530707, -
199.6083389, -241.874158, -288.8509362, -340.7099574, -397.49874, -459.4727811, -2572.3707797])
    AQci = np.array([-0.4993343, -7.4324257, -14.6169075, -24.5911252, -37.764881, -54.4870115, -74.925731, -99.5502035, -161.8530707, -
199.6412673, -241.9227443, -288.9183398, -340.7966949, -397.6105804, -459.6122531, -2572.5002195])
    ADz = np.array([-0.4993343, -7.4324257, -14.5981502, -24.5676436, -37.7414646, -54.4681736, -74.906967, -99.5356991, -161.8530707, -
199.6299162, -241.9051571, -288.8974701, -340.7768833, -397.5886211, -459.5921497, -2572.4843204])
    ATz = np.array([-0.4998212, -7.4327053, -14.6011854, -24.5765767, -37.7595607, -54.4986473, -74.9592941, -99.6121061, -161.858038, -
199.6370122, -241.9152723, -288.913275, -340.800759, -397.6283261, -459.6473317, -2572.6466854])

    Alarge = ATz - ADz
    Mlarge = MP2tz_m - MP2dz_m
    DHO = np.array([51.63, 37.69, 76.48, 136.2, 169.98, 112.53, 58.99, 18.47, 25.69, 34.87, 78.23, 106.6, 75.42, 65.66, 28.59, 28.2])
    DHT = np.array([1.01, 1.1, 0.46, 0.29, 0.25, 1.04, 1.04, 1.05, 1.54, 1.19, 1.08, 0.76, 1.28, 1.05, 1.1, 0.0])
    SO = np.array([0.0, 0.0, 0.0, -5e-05, -0.00014, 0.0, -0.00036, -0.00061, 0.0, 0.0, -0.00034, -0.00068, 0.0, -0.00089, -0.00134, -0.0056])
```

```
Atotal = np.array(
    [-0.4998212, -7.4327053, -14.619942700000001, -24.6000583, -37.782977100000004, -54.517485199999996,
     -74.97805810000001, -99.6266105, -161.858038, -199.6483633, -241.9328595, -288.9341447, -340.82057060000005,
     -397.6502854, -459.6674351, -2572.6625845000003])

Ea = np.dot(COUNT_A, Atotal)
Em = HF + QCISDT - HF + MP2tz_m - MP2dz_m + ZPE

features = np.array([H,Li, Be, B, C, N, O, F, Na, Mg, Al, Si, P, S, Cl, Br,
              QCISDT - HF,
                QCISDT - Em,
                HF - Em,
                MP2tz_m - MP2dz_m,
                Ea - QCISDT,
                Ea - HF,
                Ea-Em,
                CorrT - ZPE,
                np.dot(COUNT_A,SO),
                np.dot(COUNT_A,Alarge) - Mlarge,
                np.dot(COUNT_A,AQci) - QCISDT,
                np.dot(COUNT_A,DHO),
                np.dot(COUNT_A,DHT)])


#feature mean and standard deviation values for normalization
means = np.array(
    [3.975806451612903, 0.020161290322580645, 0.008064516129032258, 0.008064516129032258, 2.274193548387097,
     0.1975806451612903, 0.43951612903225806, 0.38306451612903225, 0.020161290322580645, 0.004032258064516129,
     0.020161290322580645, 0.056451612903225805, 0.05241935483870968, 0.125, 0.31048387096774194,
     0.056451612903225805, -0.6646262568548382, 0.13244024314516492, 0.7970665000000033, -0.1916540536290361,
     0.9599904903225531, 0.29536423346771523, 1.0924307334677186, 0.005530306451612897, -0.0015993548387096767,
```

```
    0.056435147177415025, 1.095209396774183, 680.4325, 6.317540322580627
    ])


  # check features
  if len(features) != len(means):
    print('incorrect number of features')

  stds = np.array([3.8394465950299934, 0.16679127322537463, 0.08943981053556, 0.08943981053555948, 2.1880861783369845,
          0.4807527212405861, 0.7595965130535285, 1.1009689115614274, 0.16679127322537474,
          0.06337190986089386, 0.16679127322537537, 0.2634271446945334, 0.32576552761185296,
          0.37634168586024197, 0.8778013726008058, 0.27831345043124794, 0.3497138231286686,
          0.1132359527324972, 0.43110045714460976, 0.11402466999030741, 0.7990698265268341,
          0.6398880136545518, 0.7901816082335713, 0.00182962224938435, 0.001959600330356845,
          0.03390292407843381, 0.8086434513671416, 510.21349050781606, 3.62329815423781]
          )

  # Weight and biasses array values
  W1 = np.array([[0.15279584, -0.49300611, -0.256201, -0.19260235, -0.80286676, 0.11898281, 0.38798603, -0.24671797],
[0.05107291, 0.26139247, -0.29743338, -0.69892091, -0.4979133, 0.14163479, 0.18213876, -0.09794535],
[-1.13174725, -0.49579713, -0.05479019, -0.0041899, -0.11947251, 0.42953011, 0.16081785, 0.6698038],
[-0.23394266, -0.41617253, -0.17044263, 0.31362015, -0.04318217, -0.67216635, -0.51111943, 0.45174831],
[0.39252526, -0.53488594, 0.69097239, -0.2458407, 0.30446494, 0.68172467, -0.14264289, 0.05229704],
[-0.61149782, -0.24314027, 0.64152557, 0.69410592, -0.88662022, 0.21420778, 0.3702032, -0.42527196],
[0.54782975, -0.45841339, -0.6864652, -0.03641113, 0.5125621, -0.78486562, 0.50175869, 0.28993711],
[-0.19231635, 0.51193422, -0.40665853, -0.30049878, -0.18885423, 0.06396595, 0.22723842, 0.26477793],
[-0.73149198, 0.19096626, -0.22009411, 0.20013055, -0.89829975, -0.60154045, 0.24520664, 0.36496225],
[0.06936359, 0.2172849, 0.0564525, -0.04989968, -0.71113878, -0.84102529, 0.15556304, 0.05649741],
[-0.22334957, 0.06816623, 0.02564686, -0.09618479, 0.15697746, -0.81707585, -0.15421286, 0.48999551],
[0.2812053, -0.0906023, -0.02030919, -0.68773854, 0.21346472, -0.35213536, -0.41203019, -0.20239191],
[-0.64076591, -0.24927416, -0.02902854, -0.2082835, 0.79092187, 0.45304403, 0.27484512, -0.70234656],
```

[0.28718832, 0.06715905, -0.69077492, 0.30011573, 0.34617719, -0.16180053, -0.8150025, 0.10899169],
[0.37255698, -0.03356663, -0.51635766, 0.00452637, -0.08957727, -0.6363641, 0.33964875, 0.41424891],
[-1.07141984, 0.37410334, 0.41652063, 0.45230243, 0.19333172, 0.22961046, 0.33175969, 0.02301018],
[-0.3117235, 0.23828202, 0.47364193, 0.47101447, 0.18953364, -0.07551364, -0.28571317, -0.25138023],
[-0.13897307, 0.01609158, -0.28702849, 0.13873024, 0.17541191, 0.10160437, -0.09301238, -0.03569725],
[0.09068225, -0.07130322, -0.45974851, -0.25916913, 0.23924848, 0.04021548, 0.47227228, -0.17888369],
[0.28817871, 0.04725849, 0.35147953, 0.16714841, 0.18922983, 0.36790776, -0.01332823, 0.11424513],
[0.45310065, -0.22272657, 0.37221798, 0.07246316, 0.18866926, -0.12462211, 0.15052891, -0.46412584],
[0.06783593, 0.14960088, 0.41286066, 0.28946945, -0.08598652, 0.4156605, 0.11612078, -0.60340011],
[0.68958682, -0.44938856, 0.45964342, -0.4281292, -0.27935681, -0.1443063, -0.22102104, -0.13592754],
[-0.30594692, -0.09901322, 0.02956095, -0.58971494, -0.28966704, -0.11275185, 0.02854693, 0.47508511],
[0.50669664, 0.14730932, 0.49429604, 0.45237982, -0.11219723, 0.35267526, -0.52274925, -0.10273039],
[-0.54980248, -0.48995963, 0.23849055, -0.19124123, 0.99357432, 0.55397433, -0.46348959, -0.49554828],
[0.16681547, -0.6571216, 0.06724156, -0.10945461, -0.06024517, 0.63732123, -0.17974372, -0.10785165],
[0.0463931, -0.1259068, 0.06100672, 0.30587947, 0.25799707, 0.57061636, 0.20616157, 0.14168137],
[0.58692491, -0.26435164, -0.07580793, 0.08332785, -0.28028762, 0.25619096, 0.54284, 0.19739181]])

W2 = np.array([[-0.47094628, 0.06341944, -1.06970298, -0.355544, 0.71800631, 0.47703326, 0.30097884, 0.40842867], [0.42151234, -0.59015387, 0.45232219, 0.28826818, 0.80009449, -0.11049102, -0.13628347, 0.01226466], [-0.62264037, 1.12400711, -0.15616788, 0.606152, -0.62852037, 0.06840793, -0.53006029, 0.64516121], [-0.25432545, 0.58935416, -0.01618273, -0.2205652, 0.50275666, 0.79728109, 0.4315877, 0.09786485], [0.18458554, 0.88178378, -0.93114924, -0.32751119, -0.06482195, -0.36161977, -0.36388984, 1.11801946], [-0.60675043, 0.2227504, -0.32721928, 0.31710216, 0.29092053, 0.50535607, -0.56708175, 0.81003249], [0.01151345, -0.26466098, 0.69962484, -0.26699916, 0.05931512, 0.2292653, 0.56255502, -0.53758991], [-0.59391481, -0.37994707, -0.04867595, 1.09199262, 0.19132467, -0.18026067, -0.466539, -0.97310281]])

b2 = np.array([0.019129831343889236, 0.03484582528471947, 0.030147641897201538, -0.0811561793088913, 0.4378417134284973, 0.39375823736190796, 0.28120163083076477, 0.396379679441452])

W3 = np.array([[0.25310424, -1.33569312, -1.00375938, 0.50361013], [-0.74450618, 1.12472415, -0.88995057, -0.29390097], [1.04193759, -0.20940216, -0.18388689, -0.9885022], [0.36281031, 0.68698442, 0.80767906, -1.48471534], [0.34108216, -0.66576076, 0.16649511, 0.5576039],

```
[-0.48167717, -0.41210717, 0.32237419, 0.84127247], [-0.72787881, 0.07375436, 1.12142539, 0.25874546], [-1.07132018, 0.66080832, -
0.99092692, 0.16621274]])

 b3 = np.array([-0.2928304076194763, -0.27825599908828735, 0.13551919162273407, 0.31876903772354126])


 #optimized parameters a,b,c and d for the four groups
 params = [[0.9627024436558981, 0.970629077219819, 1.3913215125987235, 1.399096056273976],
       [0.8596441561643315, 0.9809995004382951, 1.3625464659499176, 1.2372916425047151],
       [1.0028021428538798, 1.0308436034783857, 1.0790705157613856, 1.0761610864622386],
       [1.0007488440257408, 1.0413446918728706, 1.1809631227119575, 1.141013738667953]]

 #activation Relu
 def relu(x):
   v = []
   for i in x:
     if i < 0:
       v.append(0)
     else:
       v.append(i)
   return np.array(v)

 #activation softmax
 def softmax(x):
   exp_x = np.exp(x - np.max(x))
   return exp_x / np.sum(exp_x)

 #normalize feature array
 normalized = np.array((np.array(features)-np.array(means))/np.array(stds))

 # make parameter prediction using the ANN model
```

```
        # make parameter prediction using the ANN model
        pred = softmax(relu(relu(normalized.dot(W1)).dot(W2) + b2).dot(W3) + b3)

        #average parameters
        ave = [sum(z[0] for z in [np.array(params[x]) * pred[x] for x in range(4)]),
            sum(z[1] for z in [np.array(params[x]) * pred[x] for x in range(4)]),
            sum(z[2] for z in [np.array(params[x]) * pred[x] for x in range(4)]),
            sum(z[3] for z in [np.array(params[x]) * pred[x] for x in range(4)])]

        print('parameters a, b, c, d :',ave)

        a,b,c,d = ave[0], ave[1], ave[2], ave[3]
        ea = np.dot(COUNT_A, Ahf + (AQci - Ahf) * a + (ATz - ADz) * c + SO)

        # Calculate em
        em = HF + (QCISDT - HF) * b + (MP2tz_m - MP2dz_m) * d

        # Calculate dh0 and dht
        dh0 = np.dot(COUNT_A, DHO)
        dht = np.dot(COUNT_A, DHT)

        # Calculate enthalpy of formation at 0K
        enthalpy = dh0 - (ea - (em + ZPE)) * 627.5095

        # Calculate enthalpy of formation at 298K
        D298 = enthalpy + (CorrT - ZPE) * 627.5095 - dht

        print('Atomic Energy = ', ea, '\nMolecular Energy = ', em,  '\nEnthalpy(298K) = ', D298)

#example
cal_parameters(0, 0, 0, 0, 0, 0, 0, 4, 0, 0, 0, 1, 0, 0, 0, 0, -686.9960109, -687.9299736, -687.9025701, -688.2951238, 0.012672, 0.018575)
```
